# Different Roles of the Mevalonate and Methylerythritol Phosphate Pathways in Cell Growth and Tanshinone Production of *Salvia miltiorrhiza* Hairy Roots

**DOI:** 10.1371/journal.pone.0046797

**Published:** 2012-11-29

**Authors:** Dongfeng Yang, Xuhong Du, Xiao Liang, Ruilian Han, Zongsuo Liang, Yan Liu, Fenghua Liu, Jianjun Zhao

**Affiliations:** 1 College of Life Science of Zhejiang Sci-Tech University, Hangzhou, China; 2 College of Life Science of Northwest A&F University, Yangling, China; 3 Xinxiang University, Xinxiang, China; 4 Tianjin Tasly Modern TCM Resources Co., Ltd., Tianjin, China; 5 College of Medicine of NingXia Teachers University, Yinchuan, China; Instituto de Biología Molecular y Celular de Plantas, Spain

## Abstract

*Salvia miltiorrhiza* has been widely used in the treatment of coronary heart disease. Tanshinones, a group of diterpenoids are the main active ingredients in *S. miltiorrhiza*. Two biosynthetic pathways were involved in tanshinone biosynthesis in plants: the mevalonate (MVA) pathway in the cytosol and the methylerythritol phosphate (MEP) pathway in the plastids. The 3-hydroxy-3-methylglutaryl coenzyme A reductase (HMGR) is the rate-limiting enzyme of the MVA pathway. The 1-deoxy-D-xylulose 5-phosphate synthase (DXS) and 1-deoxy-D-xylulose 5-phosphate reductoisomerase (DXR) are the key enzymes of the MEP pathway. In this study, to reveal roles of the MVA and the MEP pathways in cell growth and tanshinone production of *S. miltiorrhiza* hairy roots, specific inhibitors of the two pathways were used to perturb metabolic flux. The results showed that the MVA pathway inhibitor (mevinolin, MEV) was more powerful to inhibit the hairy root growth than the MEP pathway inhibitor (fosmidomycin, FOS). Both MEV and FOS could significantly inhibit tanshinone production, and FOS was more powerful than MEV. An inhibitor (D, L-glyceraldehyde, DLG) of IPP translocation strengthened the inhibitory effects of MEV and FOS on cell growth and tanshinone production. Application of MEV resulted in a significant increase of expression and activity of HMGR at 6 h, and a sharp decrease at 24 h. FOS treatment resulted in a significant increase of *DXR* and *DXS* expression and DXS activity at 6 h, and a sharp decrease at 24 h. Our results suggested that the MVA pathway played a major role in cell growth, while the MEP pathway was the main source of tanshinone biosynthesis. Both cell growth and tanshinone production could partially depend on the crosstalk between the two pathways. The inhibitor-mediated changes of tanshinone production were reflected in transcript and protein levels of genes of the MVA and MEP pathways.

## Introduction

**Figure 1:**
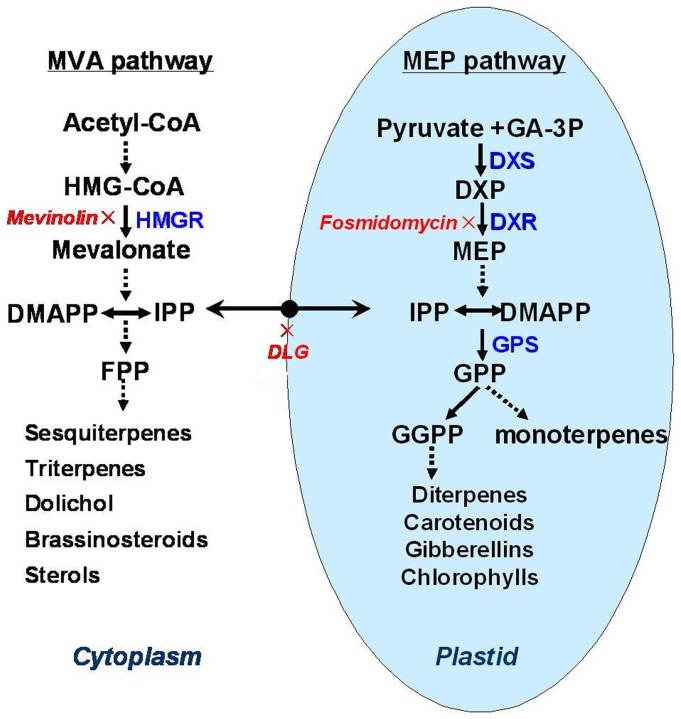
The mevalonate (MVA) and methylerythritol phosphate (MEP) pathways in biosynthesis of terpenoid. HMGR, 3-hydroxy-3-methylglutaryl coenzyme A reductase; DMAPP, dimethylallyl pyrophosphate; IPP, isopentenyl diphosphate; FPP, farnesyl diphosphate; DLG, D, L-glyceraldehyde; GA-3P, glyceraldehyde 3-phosphate; DXP, 1-deoxy-D-xylulose 5-phosphate; DXS, 1-deoxy-D-xylulose 5-phosphate synthase; DXR, 1-deoxy-D-xylulose 5-phosphate reductoisomerase; MEP, 2-C-methyl-d-erythritol-4-phosphate; GGPP, Geranylgeranyl diphosphate; GPP, Geranyl diphosphate; GGPPS, Geranylgeranyl diphosphate synthase.

It is well known that terpenoids are biosynthesized via two pathways in plants: the mevalonate (MVA) pathway in the cytosol [1] and the methylerythritol phosphate (MEP) pathway in the plastids [2] (Figure 1). The 3-hydroxy-3-methylglutaryl coenzyme A reductase (HMGR) is the rate-limiting enzyme of the MVA pathway, and mevinolin (MEV) is a highly specific inhibitor of HMGR [1]. The 1-deoxy-D-xylulose 5-phosphate synthase (DXS) and 1-deoxy-D-xylulose 5-phosphate reductoisomerase (DXR) are the key enzymes of the MEP pathway, and fosmidomycin (FOS) is an inhibitor of DXR [2]. The MVA pathway generally supplies precursors for production of sesquiterpenes, triterpenes, dolichol and brassinosteroids. The MEP pathway generally supplies precursors for the biosynthesis of diterpenoids, carotenoids, gibberellins and chlorophylls [3],[4]. Only the MEP pathway in most eubacteria is used to supply precursors for terpenoid biosynthesis, and only the MVA pathway in fungi and animals is used. In plants, however, both the MVA and MEP pathways are used for terpenoid production [3]. Yet, the crosstalk between the two pathways in plants is still unclear. Some exchanges of IPP between the cytoplasm and plastids did appear to occur, although with low efficiency [3]. Some evidence indicated that IPP exchanges were probably in both direction, whereas, the delivery of precursor from the plastids to the cytosol seemed to operate more readily [5].

**Figure 2:**
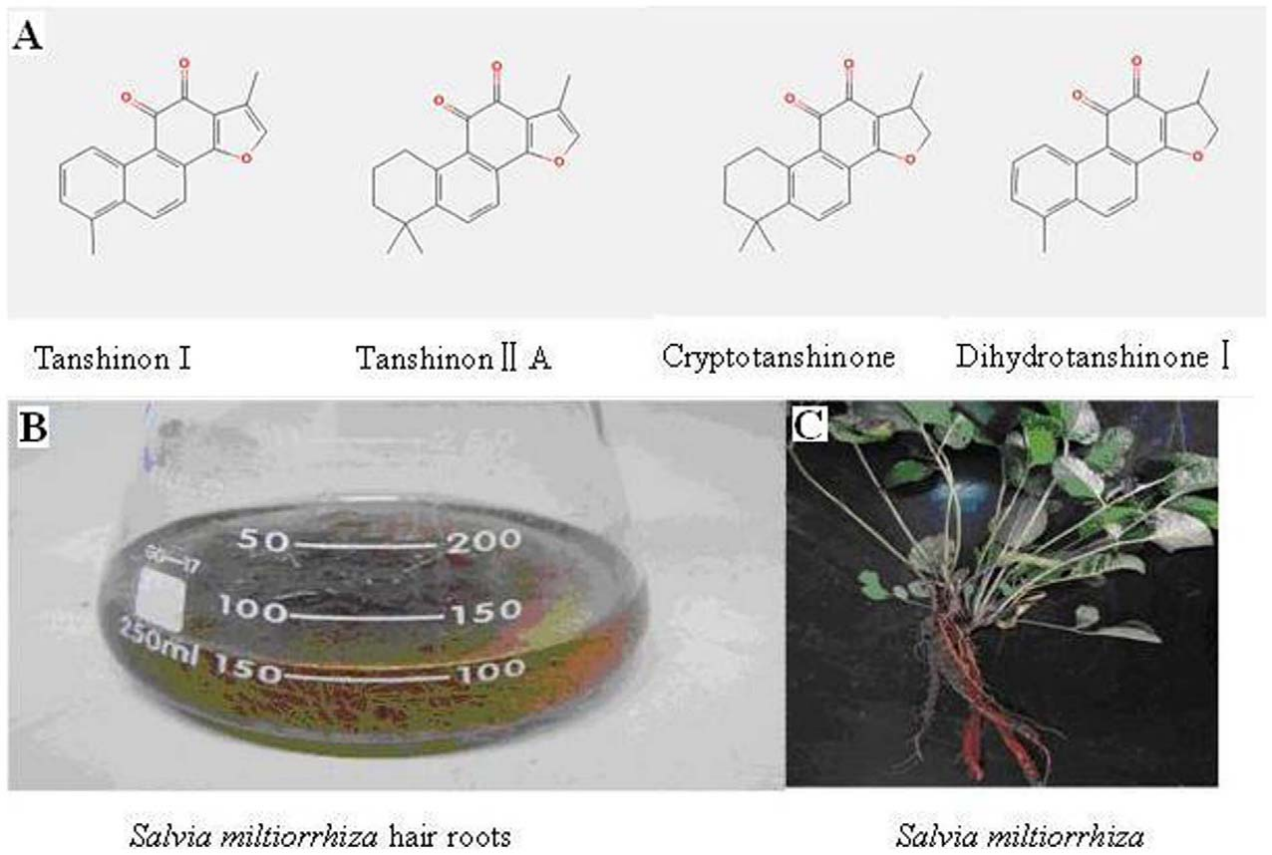
Tanshinones and *Salvia miltiorrhiza*. (A) Chemical structures of tanshinone I, cryptotanshinone, dihydrotanshinone I and tanshinone II A in *Salvia miltiorrhiza*; (B) *S. miltiorrhiza hairy* root culture; (C) The whole plant of *S. miltiorrhiza*.

*Salvia miltiorrhiza* is a famous Traditional Chinese Medicine (TCM) because of its excellent performance in the amelioration of microcirculatory disturbance [6]. Tanshinones, a group of terpenoids including tanshinone I, cryptotanshinone, dihydrotanshinone I and tanshinone II A are the main active ingredients in *S. miltiorrhiza* (Figure 2A). The involvement of the MVA and the MEP pathways in tanshinone biosynthesis has been suggested [7], [8], [9], [10]. Over-expression of HMGR and DXS in transgenic hairy root lines could significantly enhance the production of tanshinones [9]. Inhibition of HMGR and DXR activities also leaded to a decrease of tanshinone production [8]. It was previously suggested that tanshinone accumulation induced by yeast extracts and Ag^+^ was mainly synthesized via the MEP pathway, but could depend on the crosstalk between the two pathways [8]. However, our knowledge about contributions of the MVA and MEP pathways to cell growth and tanshinone biosynthesis of *S. miltiorrhiza* hairy roots is far from complete.

D, L-glyceraldehyde (DLG) is an inhibitor of IPP translocation between the cytoplasm and plastids [4], [11]. In this study, mevinolin and fosmidomycin were used to inhibit the MVA and MEP pathways, and DLG was used to block IPP translocation in *S. miltiorrhiza* hairy roots. The effects of these inhibitors on the hairy root growth and tanshinone production were investigated. Simultaneously, gene expression and enzyme activity involved in the MVA and the MEP pathways were further detected to elucidate regulation mechanism of tanshinone biosynthesis and the crosstalk between the MVA and MEP pathways

## Results

### Effects of the pathway inhibitors on cell growth of *S. miltiorrhiza* hairy roots

**Figure 3:**
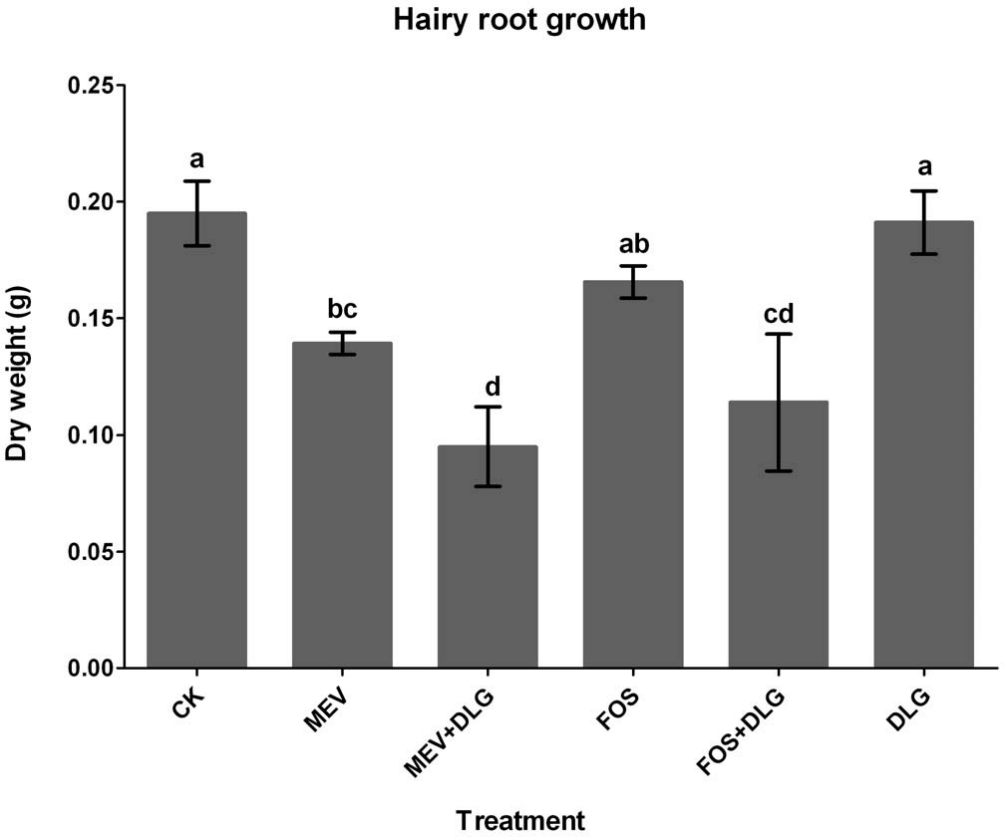
Effects of mevinolin, fosmidomycin and D, L-glyceraldehyde on cell growth of *S. miltiorrhiza* hairy roots. CK, the control; MEV, mevinolin; FOS, fosmidomycin; DLG, D, L-glyceraldehyde. Different letters indicate significant difference at *p*≤0.05 using Duncan's multiple range test. Means ± standard deviation (S.D.) (*n = 3*) are shown.

We initially measured the growth of *S. miltiorrhiza* hairy roots under inhibitor treatments. Concentrations of 1 mM DLG, 10 µM MEV and 150 µM FOS were chosen according to the preliminary experiments (data not shown). As shown in Figure 3, the hairy root growth was significantly inhibited by 10 µM MEV, and decreased by 34% when compared with the control. Treatments with 150 µM FOS resulted in a 15% decrease of the hairy root growth. These results indicated that both the MVA and MEP pathways were involved in the growth of *S. miltiorrhiza* hairy roots. However, the MVA pathway probably played a more important role in the hairy root growth than the MEP pathway. Although treatments with DLG alone hardly affected the hairy root growth, it dramatically strengthened the inhibitory effects of MEV and FOS. The hairy root growth was reduced to 48% of the control level by MEV+DLG treatment and to 58% of the control level by FOS+DLG treatment. It suggested that the growth of *S. miltiorrhiza* hairy roots could partially depend on the crosstalk between the MVA and MEP pathways.

### Effects of the pathway inhibitors on tanshinone accumulation in *S. miltiorrhiza* hairy roots

**Figure 4:**
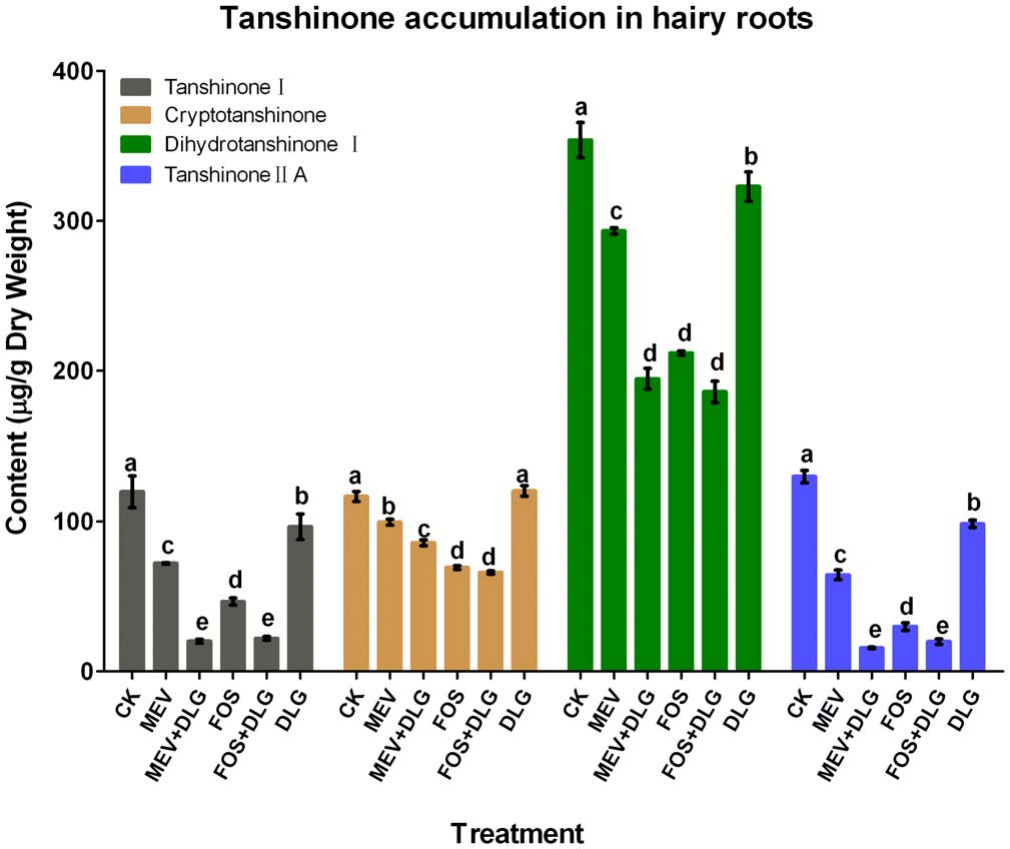
Effects of mevinolin, fosmidomycin and D, L-glyceraldehyde on tanshinone content in *S. miltiorrhiza* hairy roots. CK, the control; MEV, mevinolin; FOS, fosmidomycin; DLG, D, L-glyceraldehyde. Different letters indicate significant difference at *p*≤0.05 using Duncan's multiple range test. Means ± standard deviation (S.D.) (*n = 3*) are shown.

Accumulations of four tanshinones including tanshinone I, cryptotanshinone dihydrotanshinone I and tanshinone II A in *S. miltiorrhiza* hairy roots were determined by HPLC in this study. Treatments with MEV and FOS resulted in significant decreases of tanshinone accumulation in the hairy root as shown in Figure 4. Contents of tanshinone I, cryptotanshinone dihydrotanshinone I and tanshinone II A in the hairy root were reduced to 60, 85, 83 and 50% of the control levels by MEV, respectively. This suggested the involvement of the MVA pathway in tanshinone biosynthesis. In FOS treatment, contents of four tanshinones in *S. miltiorrhiza* hairy roots were reduced to 39, 59, 60 and 23% of the control levels, respectively. It indicated that the MEP pathway also participated in tanshinone biosynthesis. Contents of four tanshinones in FOS treatment were just 64, 70, 72 and 46% of those in MEV treatment, which suggested that FOS was more effective to inhibit tanshinone production. Those results indicated that the MEP pathway probably played a more important role in tanshinone production than the MVA pathway. By blocking IPP translocation between the cytoplasm and plastids with DLG alone, contents of tanshinone I, dihydrotanshinone I and tanshinone II A in *S. miltiorrhiza* hairy roots were just slightly reduced and that of cryptotanshinone was hardly affected. Interestingly, treatments with MEV+DLG or FOS+DLG resulted in more serious decreases of tanshinone production than treatments with MEV or FOS alone. DLG was more powerful to strengthen the inhibitory effect of MEV than to that of FOS. Accumulations of tanshinone I, cryptotanshinone, dihydrotanshinone I and tanshinone II A in *S. miltiorrhiza* hairy roots were reduced to 17, 74, 55 and 12% of the control levels by MEV+DLG treatment and to 18, 57, 53 and 15% of the control levels by FOS+DLG treatment. It was inferred that tanshinone production could partially depend on the crosstalk between the two pathways.

### Effects of the pathway inhibitors on tanshinone release and tanshinone yield of *S. miltiorrhiza* hairy roots

**Figure 5:**
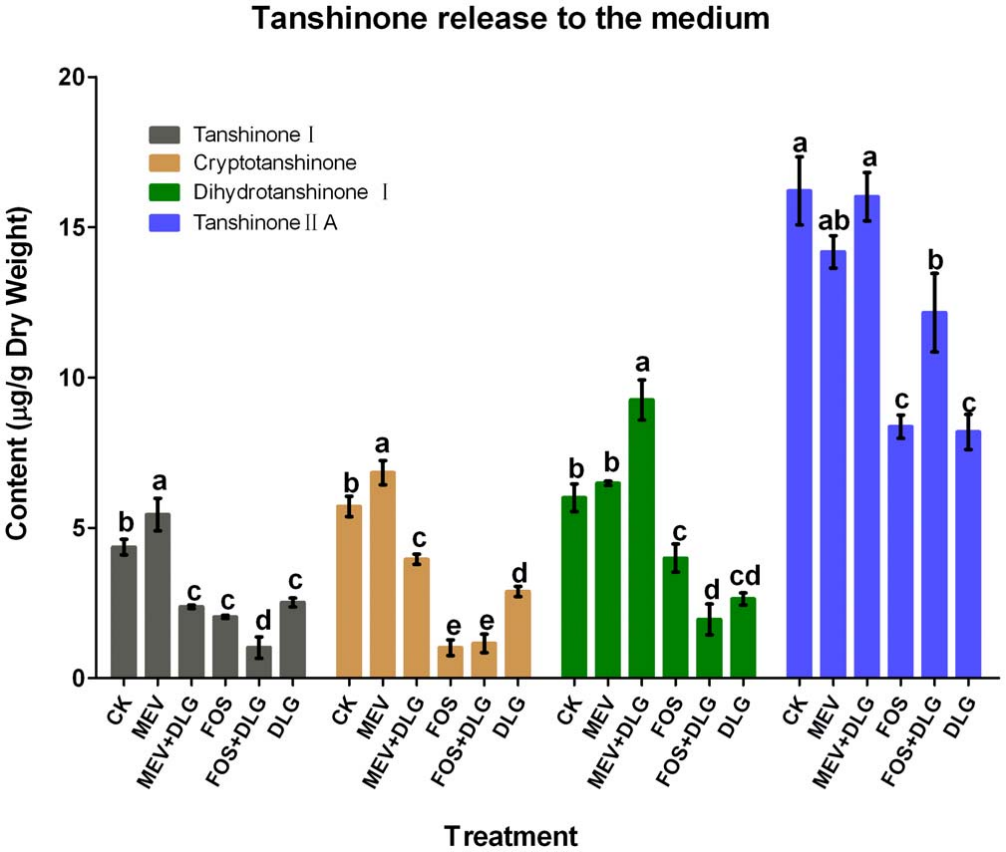
Effects of mevinolin, fosmidomycin and D, L-glyceraldehyde on tanshinone release to the medium. CK, the control; MEV, mevinolin; FOS, fosmidomycin; DLG, D, L-glyceraldehyde. Different letters indicate significant difference at *p*≤0.05 using Duncan's multiple range test. Means ± standard deviation (S.D (*n = 3*) are shown.

To investigate further effects of those inhibitors on tanshinone production, tanshinone release to the medium and tanshinone yield (the total sum of tanshinone accumulation in the hairy root and the medium) of *S. miltiorrhiza* hairy roots were also analyzed. As shown in Figure 5, tanshinone content in the medium was very low. The levels of tanshinone I, cryptotanshinone, dihydrotanshinone I and tanshinone II A contents were just 4.4, 5.7, 6.0 and 16.2 µg/g in the control. Contents of four tanshinones in the medium were decreased to 47, 18, 67 and 52% of the control levels by FOS treatment. The results indicated that FOS not only acted as an inhibitor of tanshinone production in *S. miltiorrhiza* hairy roots but also was effective to arrest tanshinone release from cells to the medium. Dihydrotanshinone I and tanshinone II A release to the medium was almost not affected by MEV, while cryptotanshinone and tanshinone I release was increased. Although DLG just slightly abolished tanshinone production in *S. miltiorrhiza* hairy roots, it resulted in a significant decrease of tanshinone release to the medium. DLG could strengthen the inhibitory effects of FOS on tanshinone I and dihydrotanshinone I release, and alleviate the decrease of tanshinone II A. The enhanced effects of MEV on cryptotanshinone and tanshinone I release to the medium were significantly inhibited by DLG.

**Figure 6:**
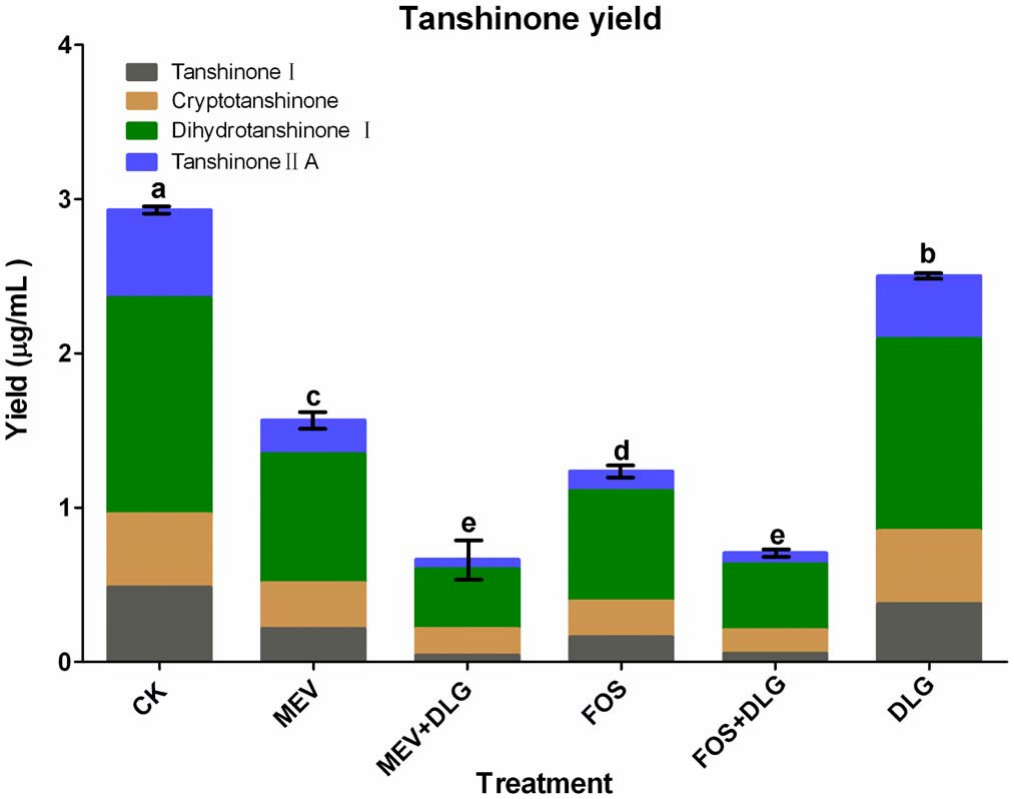
Effects of mevinolin, fosmidomycin and D, L-glyceraldehyde on tanshinone yield of *S. miltiorrhiza* hairy roots. CK, the control; MEV, mevinolin; FOS, fosmidomycin; DLG, D, L-glyceraldehyde. Different letters indicate significant difference of the total tanshinone yields of four tanshinones at *p*≤0.05 using Duncan's multiple range test. Means ± standard deviation (S.D.) (*n = 3*) are shown.

Tanshinone yield was also significantly inhibited by MEV and FOS treatments as shown in Figure 6. Yields of tanshinone I, cryptotanshinone, dihydrotanshinone I and tanshinone II A were decreased to 45, 62, 60 and 38% of the control levels by MEV, and to 33, 49, 51 and 22% f the control levels by FOS. The total yield of four tanshinones in FOS treatment was significantly lower than that in MEV treatment, which suggested that FOS was more effective to inhibit tanshinone production than MEV. Although DLG just slightly inhibited tanshinone yield, it powerfully strengthened the inhibitory effects of MEV and FOS.

### Effects of the pathway inhibitors on expression of HMGR, DXS and DXR

**Figure 7:**
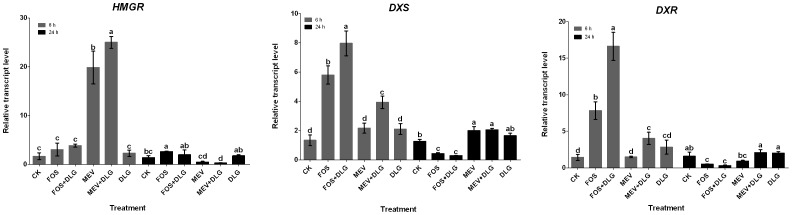
Effects of mevinolin, fosmidomycin and D, L-glyceraldehyde on the expression of genes involved in the MVA and MEP pathways. CK, the control; MEV, mevinolin; FOS, fosmidomycin; DLG, D, L-glyceraldehyde; HMGR, 3-hydroxy-3-methylglutaryl coenzyme A reductase; DXS, 1-deoxy-D-xylulose 5-phosphate synthase; DXR, 1-deoxy-D-xylulose 5-phosphate reductoisomerase. Different letters indicate significant difference at *p*≤0.05 using Duncan's multiple range test. Means ± standard deviation (S.D.) (*n = 3*) are shown.

In the MVA and MEP pathways, tens of enzymes are involved in terpenoid production. To investigate effects of inhibitors on expression of genes involved in the MVA and MEP pathways, the relative transcript abundances of *HMGR*, *DXS* and *DXR* in the hairy root treated at 6 and 24 h were detected by quantitative real time PCR. Figure 7 showed that MEV treatment resulted in a significant increase of *HMGR* expression (20-fold of the control level) at 6 h and a sharp decrease (35% of the control level) at 24 h, but it hardly affected *DXS* and *DXR* expression. In contrast, FOS significantly stimulated *DXS* and *DXR* expression (6 and 8-fold of the control levels) at 6 h and then sharply inhibited their expression (31 and 27% of the control levels) at 24 h, but it hardly affected *HMGR* expression. These results suggested that MEV mainly affected the MVA pathway and FOS principally acted on the MEP pathway. No significant effect of DLG on the expression of *HMGR, DXS* and *DXR* was observed. However, treatment with DLG not only strengthened the inhibitory effects of FOS on *DXS* and *DXR* expression, but also strengthened the inhibitory effects of MEV on *HMGR* expression at 24 h.

### Effects of the pathway inhibitors on HMGR and DXS activities

**Figure 8:**
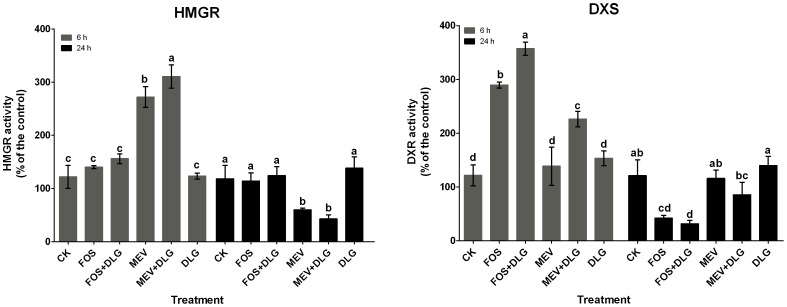
Effects of mevinolin, fosmidomycin and D, L-glyceraldehyde on activities of HMGR and DXS. CK, the control; MEV, mevinolin; FOS, fosmidomycin; DLG, D, L-glyceraldehyde; HMGR, 3-hydroxy-3-methylglutaryl coenzyme A reductase; DXS, 1-deoxy-D-xylulose 5-phosphate synthase. Different letters indicate significant difference at *p*≤0.05 using Duncan's multiple range test. Means ± standard deviation (S.D.) (*n = 3*) are shown.

To elucidate further effects of these inhibitors on the MVA and MEP pathways, activities of HMGR and DXS were determined at 6 and 24 h after treatments. As shown in Figure 8, MEV treatment led to a rapid increase of HMGR activity (271% of the control level) at 6 h and a sharp decrease (51% of the control level) at 24 h. In contrast, FOS treatment resulted in a significant increase of DXS activity (289% of the control level) at 6 h and a sharp decrease (35% of the control level) at 24 h. Treatment with DLG alone hardly affected activities of HMGR and DXS. However, the inhibitory effects of FOS and MEV were significantly strengthened by DLG at 24 h. These results were consistent with the observations at transcript level, and confirmed further that MEV mainly affected the MVA pathway and FOS primarily acted on the MEP pathway.

## Discussion

### The MVA pathway plays a major role in cell growth of *S. miltiorrhiza* hairy roots

The MVA and MEP pathways were not only responsible for production of secondary metabolites, but also involved in the biosynthesis of primary metabolites necessary for cell growth and maintenance [3], [4]. Increasing evidence indicated that mevinolin, a specific inhibitor of HMGR acted as a highly efficient plant growth inhibitor [5], [12], [13]. Root elongation of radish and *Arabidopsis* seedlings was observed to be significantly inhibited by 2.5–10 µM MEV [12], [13]. Treatment of Tobacco Bright Yellow-2 (TBY-2) cells by 5 µM MEV also led to more than 90% of cell growth reduction [5]. The similar results were observed in our experiments. It indicated that the MVA pathway played an important role in *S. miltiorrhiza* hairy root growth. Mevinolin inhibits strongly in plants the biosynthesis of sterols, which are synthesized via the MVA pathway. Accordingly, cell division is hampered by MEV, and it is not surprising that this inhibitor stops the hairy root growth. Fosmidomycin, a highly specific inhibitor of DXR led to a significant decrease of *Artemisia annua* L. growth at 100 µM [14]. The decrease of tobacco BY-2 cell growth in 20 µM FOS treatment has been observed, although it was much less efficient than that in 5 µM MEV treatment [5]. In *S. miltiorrhiza* hairy root culture, we observed that the cell growth was justly slightly arrested by FOS. These results indicated that the MEP pathway was also involved in cell growth of *S. miltiorrhiza* hairy roots, but probably played a minor role. Previous reports inferred that the cell growth was hardly influenced by 1 mM DLG during the whole period of suspension cultures of *Taxus chinensis* var. *mairei* [11]. The results held true for *S. miltiorrhiza* hairy roots. However, the inhibitory effects of FOS and MEV were largely strengthened by DLG. These observations suggested that cell growth of *S. miltiorrhiza* hairy roots partially depended on the crosstalk between the MVA and MEP pathways, although the MVA pathway played a major role.

### The MEP pathway plays a major role in the biosynthesis of tanshinones

Increasing evidence indicated that contributions of the MVA and MEP pathways to the diverse terpenoids were dissimilar [15], [16], [17]. For example, gibberellins were predominantly synthesized through the MEP pathway, whereas the MVA pathway played a major role in the biosynthesis of campesterol [17]. While the MVA pathway was responsible for sterols biosynthesis, the MEP pathway provided the precursors for carotenoids and chlorophylls production [15]. Ge Xiuchun and Wu Jianyong tried to elucidate roles of the two pathways in tanshinone biosynthesis, and suggested that tanshinone accumulation induced by yeast extract and Ag^+^ was mainly synthesized via the MEP pathway, but could depend on the crosstalk between the MVA and MEP pathways [8]. In transgenic *S. miltiorrhiza* lines, DXS showed much more powerful pushing effect than HMGR in tanshinone biosynthesis, which indicated that genetic manipulation of the MEP pathway resulted in significantly higher yields than the MVA pathway enhancement [9]. This presumption was substantiated in our experiments. Although both MEV and FOS were effective to inhibit tanshinone production, FOS was more powerful than MEV. It indicated that the MEP pathway probably played a major role in tanshinone biosynthesis.

Previously it was shown that 5 µM MEV led to a rapid increase of HMGR activity in tobacco BY-2 cells [5]. The transcripts accumulations of genes involved in carotenoid biosynthesis including *DXR* and *DXS* were significantly induced in tomato by 100 µM FOS [18]. FOS also led to a rapid accumulation of DXR protein at 100 µM [19]. These observations held true for the inhibitor-treated *S. miltiorrhiza* hairy roots. Gene expression and enzyme activity involved in the MVA pathway in *S. miltiorrhiza* hairy roots were dramatically stimulated by MEV, and those involved in the MEP pathway were up-regulated by FOS at 6 h. The eventual increase of gene expression and enzyme activity might be caused by a feedback mechanism to restore HMGR and DXR to normal levels [5], [18], [19]. However, a sharp decrease of gene expression and enzyme activity were observed at 24 h, which probably caused significant reduction of tanshinone production. These results indicated MEV specifically acted on the MVA pathway and FOS specifically acted on the MEP pathway.

All living organisms could produce terpenoid, but only one pathway is used to synthesize the isoprenoid precursors by most organisms. In plants, however, the two pathways are used for terpenoid biosynthesis [3]. Although the evolutional mechanism of the phenomenon is still unknown, the crosstalk between the two pathways has been confirmed [5], [15], [16], [17], [20], [21]. A unidirectional proton symport system for the export of IPP and geranyl diphosphate (GPP) from cytoplasm to plastids has been proved [20]. The experiment of feeding mevalonic acid lactone and 1-deoxy-D-xylulose [22] with stable isotopes revealed that both the MVA and MEP pathways supplied IPP for terpenoid biosynthesis in *Daucus carota* L. and a unidirectional import of IPP from the plastids to cytoplasm was also required [21]. However, growing evidence suggested that the IPP exchange was probably in both directions [5], [15], [17]. DLG was an inhibitor of IPP translocation. It has been reported that taxol production in suspension cultures of *Taxus chinensis* var. *maire* was inhibited by 1 mM DLG at the late growth phase, and it suggested that the decrease of taxol production by DLG was probably caused by the decrease of IPP translocation but not by the damages of DLG to the primary metabolism [11]. In DLG-treated *S. miltiorrhiza* hairy roots, tanshinone production was just slightly reduced, and gene expression and enzyme activity involved in the two pathways were hardly affected when compared with the control. The slight effects of DLG were probably due to the compensation of IPP supply from the MVA and MEP pathways. However, DLG could significantly strengthen the inhibitory effects of MEV and FOS on tanshinone biosynthesis. Although tanshinones were predominantly derived from the MEP pathway in plastids, their accumulation partially depended on IPP supply from the MVA pathway. These results suggested that the crosstalk between the two pathways probably played an important role in tanshinone biosynthesis.

## Conclusions

In summary, our results suggested that the MVA pathway played a major role in cell growth of *S. miltiorrhiza* hairy roots. Both the MVA and MEP pathways were involved in tanshinone production, while the MEP pathway was probably the main source of precursors in the biosynthesis of four tanshinones. Both cell growth and tanshinone production could partially depend on the crosstalk between the two pathways. The inhibitor-mediated changes were reflected in transcript and protein levels of genes of the MVA and MEP pathways.

## Methods

### Hairy root culture and application of inhibitors

*S. miltiorrhiza* hairy roots were derived after the infection of plantlets with *Agrobacterium rhizogenes* bacterium (ATCC15834). Experiments in this study were carried out in a 250 mL shake-flask on an orbital shaker running at 110–120 rpm and at 25°C in darkness [23]. Each flask was filled with 50 mL liquid medium and inoculated with 0.3 g fresh hairy roots from a 3-week-old shake-flask culture. The liquid medium was made of hormone-free MS medium with 30 g/L sucrose but without ammonium nitrate as previously described [23].

Stock solutions of D, L-glyceraldehyde (Sigma, USA) and fosmidomycin sodium salt (Santa Cruz biotechnology, USA) were prepared in distilled water and sterilized by filtration (0.22 µm membrane). Mevinolin (Sigma, USA) was previously converted to the water-soluble sodium salt as described [24]. Treatments with inhibitors were performed on day 18 post inoculation. Concentrations of MEV, FOS and DLG were at 10 µM, 150 µM and 1 mM, respectively. The equal volume of distilled water was added to the hairy root culture as the control. All treatments were performed in triplicate. Hairy roots were harvested from the culture medium on day 6 after treatments and dried at 45°C in an oven until constant weight.

### Tanshinone extraction and HPLC analysis

HPLC analysis was performed using a Waters (Milford, MA, USA) system with a binary pump and Photodiode Array Detector (DAD) as previously described [25]. A SunFire C18 column (250 mm×4.6 mm, 5 µm) was used.

The dried hairy roots were comminuted to powder in a mortar and sieved through a 0.45-mm screen. The powder (0.1 g) was extracted ultrasonically with 2 mL of methanol-water solution (7∶3) for 45 min. The extracts were centrifuged at 10,000 rpm for 15 min and filtered through a 0.45-µm Millipore filter. Tanshinone in the medium was extracted by equal volume of chloroform for three times, and the extracts were evaporated, then the sample was redissolved in 1 mL methanol and filtered through a 0.45-µm Millipore filter

### Quantitative real-time PCR analysis

Hairy roots 24 h after treatment were homogenized in liquid nitrogen to fine powder. The total RNA was extracted by RNAisoTM Plus (Takara,Tokyo, Japan). The first strand cDNA was synthesized from 500 ng total RNA using the reverse transcription PCR system according to the manufacturer's protocol of PrimeScript® RT reagent Kit (Takara,Tokyo, Japan). Primers used for the first strand cDNA synthesis were Oligo dT primer supplied in the Kit. The quantitative RT-PCR reactions were performed by a Bio-Rad CFX96 system (Bio-Rad, USA) with Brilliant II SYBR® Green QPCR Master Mix (Agilent, Santa Clara, USA). A total reaction volume of 25 µL included 12.5 µL Brilliant II SYBR® Green QPCR Master Mix, 1.0 µL 10 µM forward primer, 1.0 µL 10 µM reverse primer, 1.0 µL cDNA template and 9.5 µL nuclease-free PCR-grade water. The conditions were: pre-denaturation at 95°C for 10 min; 40 cycles of denaturation at 95°C for 30 sec; annealing at 60°C for 60 sec and collection fluorescence at 72°C for 30 sec. Experiments were performed in triplicate and the results were represented by their means ± standard deviation (S.D.). β-actin was the reference gene. The expression levels of *HMGR*, *DXS* and *DXR* was detected. The primers were designed by the software Primer-Premier 5.0 (Palo Alto, Canada) (Table 1).

**Table 1:** The primers of genes used in RT-PCR.

Name	Sequence
HMGR-F	5′-GCAACATCGTCTCCGCCGTCTACA-3′
HMGR-R	5′-GATGGTGGCCAGCAGCCTGGAGTT-3′
DXS-F	5′-AGAGCGACTACGACTGCTTTGG-3′
DXS-R	5′-CAGGTAGCCAGCATTGTTCATT-3′
DXR-F	5′-CATGCGTTTGCCTATTCTGTAC-3′
DXR-R	5′-ACTAAGAACTCCGGTCATGGTG-3′
β-actin-F	5′-AGGAACCACCGATCCAGACA-3′
β-actin-R	5′-GGTGCCCTGAGGTCCTGTT-3′

### Enzyme assays

The hairy root (1.0 g) was ground with 1 mL of extraction buffer (50 mM Tris–HCl, 10 mM β-mercaptoethanol, 1% (w/v) polyvinylpyrrolidone and pH 7.5) for 5 min on ice, and then centrifuged at 14,000 rpm for 30 min. The protein concentration was determined by the Bradford method using bovine serum albumin as a standard [26]. The activities of HMGR and DXS were determined by the method as previously described [8]. The reaction mixture (50 mM Tris–HCl pH 7.0, 0.3 mM HMG-CoA, 0.2 mM NADPH, 4 mM dithiothreitol and the enzyme extract) was monitored at 340 nm (25°C) to determine HMGR activity. One HMGR enzyme unit (U/mg protein) is equivalent to the oxidation of 1 µmol of NADPH per minute. To determine DXS activity, the reaction mixture (40 mM Tris–HCl (pH 7.5), 2.5 mM MgCl_2_, 5 mM β-mercaptoethanol, 1 mM thiamin diphosphate, 10 mM sodium pyruvate, 20 mM DL-glyceraldehyde 3-phosphate, and the enzyme extract) was incubated at 37°C for 1 h and 80°C for 5 min, and followed by centrifugation at 13,000 rpm for 5 min. Then, after the supernatant was mixed with 1 mL of 10 mM 3, 5-diaminobenzoic acid in 5 M phosphoric acid and incubated at 100°C for 15 min, the fluorescence intensity of the reaction product was measured at 396 nm excitation and 510 nm emission with a fluorescence spectrophotometer. The activities of HMGR and DXS in inhibitor-treated *S. miltiorrhiza* hairy roots were calculated against those in the control, and the results were shown as folds of the control.

### Statistical analysis

All data in this work were obtained from three independent experiments with three replicates each. Statistical analysis was performed using one-way analysis of variance (ANOVA) and followed by Duncan's multiple range test with Data Processing System (DPS) for windows. The difference between treatments was considered to be statistically significant when *p* values ≤0.05.
